# 

**Published:** 1858

**Authors:** 


VOL. IV.] [NUMBER 3.

JANUARY, FEBRUARY AND MARCH, 1858.

### IOWA MEDICAL JOURNAL.

CONDUCTED BY THE FACULTY OF THE

### MEDICAL DEPARTMENT

OF THE

### IOWA STATE UNIVERSITY.

EDITORS:

J. C. HUGHES, M. D.,

Prof. of Surgery in the Medical Department of the Iowa University.

WELLS R. MARSH, M. D.

Prof. of Chemistry and Toxicology in Med. Dep't. of Iowa University.

KEOKUK, IOWA:

PRINTED AT THE JOURNAL BOOK AND JOB OFFICE.

1858.

